# Harnessing the Value of *Tripolium pannonicum* and *Crithmum maritimum* Halophyte Biomass through Integrated Green Biorefinery

**DOI:** 10.3390/md21070380

**Published:** 2023-06-27

**Authors:** Laura Sini Sofia Hulkko, Tanmay Chaturvedi, Luísa Custódio, Mette Hedegaard Thomsen

**Affiliations:** 1 AAU Energy, Aalborg University, Niels Bohrs Vej 8, 6700 Esbjerg, Denmark; 2 Centre of Marine Sciences, University of Algarve, Campus of Gambelas, 8005-139 Faro, Portugal

**Keywords:** biomass, biorefinery, halophytes, bioprospecting, bioproducts, phytochemicals

## Abstract

Bioactive extracts are often the target fractions in bioprospecting, and halophyte plants could provide a potential source of feedstock for high-value applications as a part of integrated biorefineries. *Tripolium pannonicum* (Jacq.) Dobrocz. (sea aster) and *Crithmum maritimum* L. (sea fennel) are edible plants suggested for biosaline halophyte-based agriculture. After food production and harvesting of fresh leaves for food, the inedible plant fractions could be utilized to produce extracts rich in bioactive phytochemicals to maximize feedstock application and increase the economic feasibility of biomass processing to bioenergy. This study analyzed fresh juice and extracts from screw-pressed sea aster and sea fennel for their different phenolic compounds and pigment concentrations. Antioxidant and enzyme inhibition activities were also tested in vitro. Extracts from sea aster and sea fennel had phenolic contents up to 45.2 mgGAE/gDM and 64.7 mgGAE/gDM, respectively, and exhibited >70% antioxidant activity in several assays. Ethanol extracts also showed >70% inhibition activity against acetylcholinesterase and >50% inhibition of tyrosinase and α-glucosidase. Therefore, these species can be seen as potential feedstocks for further investigations.

## 1. Introduction

**Figure 1:**
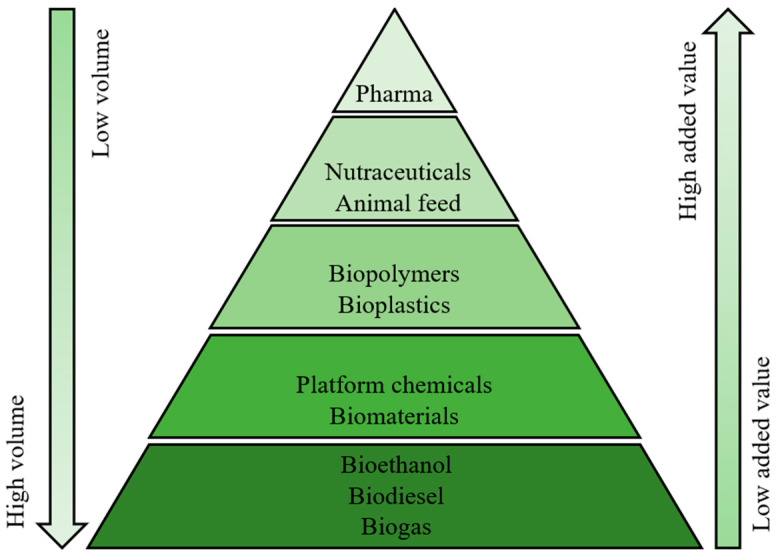
Value pyramid of bioproducts. Adapted from Stegmann et al. [8] and Zabaniotou [2].

In order to mitigate global warming and meet the set climate goals, it is necessary to transition from a linear fossil-based economy towards circular bioeconomies and sustainable agricultural intensification, which leads to an expected increase in the world’s biomass demand driven by food security, bioproducts and bioenergy [1,2,3]. Muscat et al. [4] argued that for the effective use of available biomass, producing goods for fulfilling basic human needs, and the chemical industry, due to a lack of sustainable alternatives, should have the highest priority. The need to find bio-based alternatives to various essential products highlights the importance of integrated biorefineries, where multiple products are produced together with bioenergy in a cascade system to maximize the valorization of the available biomass while also increasing the economic feasibility of the processing [2,4,5,6,7]. This concept is visualized in Figure 1. Furthermore, these multi-product systems have also been described as an important pillar and tool for circular bioeconomies [2,8]. According to Stegmann et al. [8], products targeting high-value applications, such as biopharmaceuticals and nutraceuticals, are considered to have significant potential within circular bioeconomies. Therefore, bioprospecting the feedstock for these applications can be seen as an inseparable part of future integrated biorefinery design, as maximum value creation can often be vital for process profitability. The agricultural and food processing sectors are significant producers of plant biomass, and currently, residual fractions are often used for biogas or bulk chemical production, compost, or even end up in landfill [7,9,10,11]. However, Jimenez-Lopez et al. [7] and Caldeira et al. [12] emphasize the potential of valorizing these fractions through the production of bioactive and functional compounds. Cascading biorefineries targeting products for health, food and feed, and cosmetics industries have also been at the forefront of the discussions related to, for example, algal biomass processing [13]. Due to limited freshwater resources and constrained availability of arable land due to increased soil and water salinity, biosaline agriculture focused on cultivating naturally salt-tolerant plants, i.e., halophytes, have been seen as one of the key solutions to sustainably create added value and rehabilitate marginal degraded areas [14,15,16]. Marine biomass, including coastal plants, could also contribute to nutrient uptake and recovery as a part of future blue-green bioeconomies [17].

According to the definition by Flower and Colmer [18], halophytes are plants that can grow and reproduce under salinity conditions of 200 mM NaCl or more, constituting approximately 1% of the world’s plant species. As a part of their adaptation to extreme environmental conditions, such as high salinity, drought, and strong UV radiation, halophytes produce high concentrations of protective bioactive phytochemicals. This way, plants maintain ion homeostasis and protect themselves from cellular damage and the metabolic issues caused by the production and accumulation of reactive oxygen species [19,20]. Due to this pronounced availability of bioactive compounds, halophytes have increased interest in bioprospecting for ingredients for nutraceuticals, cosmetics, and biomedicines [5,19,20,21,22,23,24]. *Tripolium pannonicum* (Jacq.) Dobrocz. (Asteraceae, syn. *Aster tripolium*, commonly known as sea aster) and *Crithmum maritimum* L. (Apicaceae, commonly known as sea fennel or rock samphire) are edible facultative halophytes used for culinary purposes and suggested species for biosaline agriculture [15,16,25,26,27]. They can be efficiently grown in hydroponic and aquaponic systems, and they have shown potential for cultivation in degraded soils and marginal land areas [25,28,29,30,31]. The extracts from these plants have been reported to have antioxidant, anti-inflammatory, antimicrobial, antiviral, antifungal, neuro-protective, anti-diabetic, and antitumor properties [21,32,33,34,35,36,37,38,39,40,41]. In folk medicine, sea fennel was used for its diuretic properties, parasite prevention, and in case of digestive issues; sea aster was used as an expectorant to relieve cough [26,41,42]. Potent essential oils from sea fennel are also suggested for pest control [43,44,45].

In the green biorefinery concept, the non-food grade fractions of plants are harvested and screw-pressed into liquid (juice) and solid (fiber residue) fractions [6,46]. This approach has been previously tested for sea aster and sea fennel, focusing on the distribution of primary metabolites [31]. Green biorefinery is commonly used for grasses and forages, where the juice is often used to produce protein and organic acids, and the solid fraction is fed to animals or used for bulk materials and biogas [47,48,49]. As the high salt content of halophytes may limit their direct use as fodder [25,50], bioactive botanical extracts rich in phytochemicals could provide another value-added stream for halophyte-based biorefinery. This approach could be desirable for edible halophytes, as it would provide not only a nutrition-rich food source but also a way to valorize the residual fractions of the plants for bioproducts after the food production period and harvesting fresh leaves for food, as the plants become unpalatable due to lignification. After the preparation of extracts, the residual lignocellulosic fibers can be used to produce biochemicals and bioenergy in more traditional biorefinery processes, as extraction may also work as a mild pretreatment of often recalcitrant lignocellulose [11,51,52,53,54,55]. In this regard, biogas production from sea aster has been previously studied [53,55,56].

**Figure 2:**
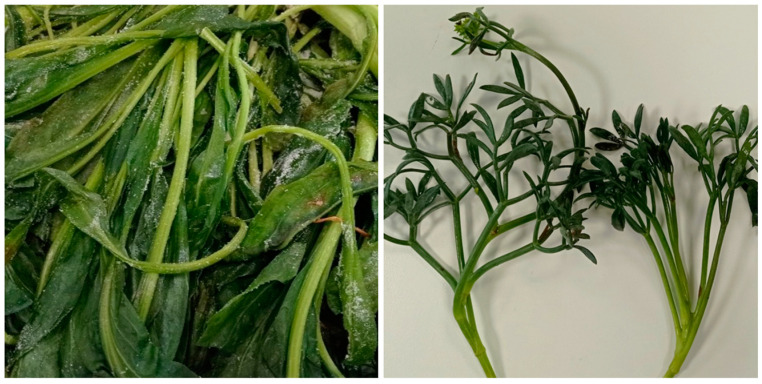
*Tripolium pannonicum* (**left**) and *Crithmum maritimum* (**right**) biomass.

This study analyzed the biological activity of sea aster and sea fennel (Figure 2) juice and extracts from fiber residue fractions to evaluate their suitability for the production of high-value, low-volume products. Non-polar extracts were used to determine fatty acid (FA) profiles, whereas juice fractions and polar extracts were analyzed for the concentration of total phenolic compounds (TPC), total flavonoids compounds (TFC), total condensed tannins (TCT), total anthocyanidins (TAC), as well as photosynthetic pigments. In addition, antioxidant properties were tested in vitro for radical scavenging activity (RSA) against 2,2-diphenyl-1-picrylhydrazyl (DPPH), 2,2′-azinobis-(3-ethylbenzothiazoline-6-sulfonic acid) (ABTS), and nitric oxide (NO), as well as iron-reducing antioxidant power (FRAP), and iron and copper chelating activities (ICA and CCA, respectively). The inhibition capabilities of juices and polar extracts were also tested in vitro against enzymes involved in neurodegenerative diseases such as Alzheimer’s disease (acetylcholinesterase and butyrylcholinesterase), hyperpigmentation (tyrosinase), type 2 diabetes mellitus (α-amylase and α-glucosidase), as well as obesity and acne (lipase). This study provides novel information about the bioactivity of sea fennel, as previous studies have heavily focused on essential oils or the fresh edible fraction of the plant. Also, it describes the bioactivity of sea aster extracts, which have not, to the best of our knowledge, been previously widely reported in the framework of bioprospecting and biorefinery.

## 2. Results and Discussion

### 2.1. Fractionation and Extraction Yields

After fractionation with a screw press, the unfiltered juice fraction corresponded to >80% of the total fresh biomass weight in both halophyte species. The composition of lignocellulosic fibers was determined from the residual material after the water extraction in terms of cellulose (glucose and cellobiose), hemicellulose (xylose and arabinose) and Klason lignin. For sea aster fibers, the lignocellulose consisted of 44.9 (5.4)% cellulose, 29.3 (0.5)% hemicellulose, and 31.2 (5.0)% Klason lignin. In sea fennel, the lignocellulose fraction contained 42.9 (0.3)% cellulose, 22.0 (0.2)% hemicellulose, and 35.1 (0.1)% Klason lignin. The dry matter (DM) content, the chemical composition of the juice and fiber fractions, and the yields of the extracts prepared from fiber residue fractions are summarized in Table 1. Extractives yields of 18.7% and 16.5% were obtained from sea aster and sea fennel fibers, respectively, using water as a solvent, whereas with ethanol, 25.9% and 13.6% sea aster and sea fennel extraction yields, respectively, were achieved. Water-soluble salts accumulated in the plant tissues are present in the extracts, and the ash content of dry sea aster and sea fennel water extracts was 22.72 (1.66)% and 27.08 (0.92)%, respectively. Similarly, in the ethanol extracts from sea aster and sea fennel, the ash content was 18.45 (1.80)% and 14.43 (4.71)%, respectively.

Even within the same species, the extraction yields may vary greatly due to intraspecific variations and depending on the solvent purity, extraction method, and potential pretreatments, for example, size reduction methods and ultrasound and microwave technologies [51]. Regarding sea fennel, Costa et al. [57] compared supercritical fluid extraction and ultrasound-assisted extraction methods to obtain ethanol extracts from wild-harvested sea fennel aerial parts and reported 11.2% and 33.2% yields with ultrasound-assisted extraction with 100 *v*/*v*% and 80 *v*/*v*% ethanol, respectively, whereas supercritical fluid extraction yielded only 4.5% of the extract. Souid et al. [24] reached 25% extract yield with three overnight macerations with 80% ethanol. Considering water-soluble sea fennel extractives, Pereira et al. [34] obtained 47.8% and 50.0% extract yields from “cup-of-tea” infusion and decoction of fresh sea fennel leaves, respectively. Similarly, Pedreiro et al. [58] achieved 36.3% and 46.1% extraction yield with infusion and decoction, respectively. Results from previous studies are significantly higher than the yield of water extract obtained from the fiber fraction, as a high fraction of water-soluble compounds is present in the green juice separated in the initial screw-press fractionation.

Yields of aqueous or ethanol extracts from sea aster have not been previously widely reported, but Wubshet et al. [39] obtained an 8.3% yield in maceration with 70% methanol. Wisznieska et al. [58] reported that salt stress increases the amount of soluble carbohydrates in sea aster biomass. Therefore, the cultivation conditions may affect the concentration of sugars detected from the juice fractions. The desired composition of the lignocellulose fraction depends on the target application, and cellulose, hemicellulose, and lignin from extractives-free residue could be potentially used to produce various value-added chemicals [50]. The ash content of juice and fiber fractions are similar to those previously reported for sea aster cultivated at 171 mM NaCl and sea fennel cultivated in non-saline hydroponics [30].

### 2.2. Fatty Acids in Non-Polar Extracts

Considering the FA profile of sea aster, the health-beneficial polyunsaturated fatty acids (PUFA) constituted 78.2% of the total FA, followed by 20.6% saturated fatty acids (SFA) and 1.2% monounsaturated fatty acids (MUFA). The main FA found was α-linolenic acid (53.2%), and the ω-6 and ω-3 ratio was 0.5. Similar results have also been reported by Duarte et al. [59], who also found PUFA to cover >70% of total FA in sea aster and a <0.5 ratio of ω-6 and ω-3. Montero et al. [38] reported the content of SFA in sea aster lipids to be 19.3%, which is comparable to obtained results. Both previous studies also report α-linolenic acid as the most pronounced FA [38,59].

The total FA from sea fennel n-hexane extract constituted 49.9% PUFA, 36.7% SFA, and 13.4% MUFA. The main FA was linoleic acid (34.4%), and the ω-6 and ω-3 ratio was 2.2. The results were similar compared to those reported by Ben Hamed et al. [60] for sea fennel cultivated in non-saline conditions in a greenhouse, the main differences being the lower amount of stearic acid (3.2% of total FA compared to obtained 6.3%) and higher amount of α-linolenic acid (24.1% of total FA compared to obtained 15.5%). The obtained ω-6 and ω-3 ratio is also comparable to the ratio of 2.7 determined by Guil-Guerrero and Rodríguez-García [61] for the neutral lipid fraction from young sea fennel leaves. Only 11.5% of oleic acid was found in vegetative plant extract, whereas the seed oil from sea fennel was previously reported to be rich in oleic acid (78% of total FA) [62].

Relative concentrations of fatty acids in n-hexane extracts from halophyte fiber residues are summarized in Table 2. In both species, the ratio of ω-6 and ω-3 FA is low, which has been linked to a reduced risk of cardiovascular diseases and cancer, and the alleviation of diseases with chronic inflammation [63]. However, the total lipids content of halophytes is often low and obtained yields for n-hexane extracts were only 2.9% and 2.2% from sea aster and sea fennel fibers, respectively. Therefore, even if the lipids are rich in health-beneficial PUFA, the role of lipids from these species in industrial biorefinery applications may not be as significant as other extracts.

### 2.3. Bioactive Compounds

The total contents of phenolic compounds and pigments determined in juice and extracts of sea aster and sea fennel are presented in Table 3. The highest concentration of TPC was found in the ethanol extract from sea fennel (64.7 mgGAE/gDM). The two species had no significant difference in the TPC content of aqueous extracts, with results being 32.3 mgGAE/gDM and 33.5 mgGAE/gDM for sea aster and sea fennel, respectively. However, extracts from sea aster fibers had a higher concentration of TFC, 5.4 mgQE/gDM and 6.6 mgQE/gDM in aqueous and ethanol extracts, respectively. Condensed tannins were only found in low concentrations from sea aster ethanol extract, whereas they were not detected in other samples. Depending on the target application, the lack of condensed tannins, also called proanthocyanidins, can be seen as an advantage, as tannins can be considered potential antinutrients due to their protein-binding properties, depending on the intake [64]. However, tannins have been previously studied for their anti-parasitic effects [65]. It was not possible to reliably determine the content of total anthocyanidins for ethanol extract, as precipitation was observed in samples. Photosynthetic pigments were found mainly in the ethanol extracts, and especially sea aster was rich in chlorophyll (3631.95 μg/gDM) and carotenoids (299.05 μg/gDM).

Meot-Duros et al. [35] found 33 mgGAE/gDM of TPC, which is similar to the result from the aqueous extract, in a water-methanol extract from sea fennel harvested from the wild in late summer. However, Pereira et al. [34] reported approximately 70.5 mgGAE/gDM TPC in sea fennel leaf extracts but also found 114.1 mg rutin equivalent/gDM TFC and 1.6 mgCE/gDM TCT; however, the extracts from sea fennel stems exhibited much lower concentrations of TPC, and phytochemicals are distributed differently to plant organs. Similarly, Mekinić et al. [37] measured greatly different amounts of TPC from sea fennel leaves (35.1 mgGAE/gDM) and stems (7.6 mgGAE/gDM). Politeo et al. [66] also reported a similar trend in aqueous sea fennel extracts.

Considering sea aster, Stankovic et al. [67] reported that a methanol extract obtained by maceration contained 144.75 mgGAE/gDM phenolic compounds and 55.43 mg rutin equivalent/gDM flavonoids. This study utilized the extract from screw-pressed fibers from the whole aerial part of the biomass instead of the whole fresh plant or selected part of plants, such as leaves, which may explain the differences in the content of phenolics. Some phytochemicals are also shown to be sensitive to different pH conditions or elevated temperatures, which, used in different extraction methods, may lead to compound instability and degradation [68]. Differences in sea fennel flavonoid content could also be explained by abiotic stresses and intraspecific variation but also by different assays used. The effect of abiotic stresses on the content of phenolic compounds is highlighted in various studies [35,64,69,70,71]. For example, salinity and exposure to heavy metals have been shown to increase the content of total phenols and antioxidant activity in sea aster [69,71,72].

Cultivation conditions and abiotic stresses also affect the content of photosynthetic pigments in plants, and Wiszniewska et al. [72] reported that the salinity stress increased the amount of chlorophyll in sea aster, whereas chlorophyll and carotenoid contents decreased when plants were exposed to heavy metals. On the contrary, Ventura et al. [71] reported an inverse relationship between cultivation salinity, and chlorophyll and β-carotene content of sea aster leaves. Duarte et al. [70] also showed that the content of chlorophyll and some carotenoids decreased when sea aster plants were exposed to heat or cold waves; however, the exposure to heat waves did not affect the content of β-carotene, lutein and certain xanthophylls. Geissler et al. [73] determined the content of chlorophyll and carotenoids in the sea aster leaf surface to be approximately 51 mg/cm^2^ and 8 mg/cm^2^, respectively. Chlorophyll is especially sensitive to degradation, and different drying methods have been shown to affect the chlorophyll concentration of sea fennel; microwave-assisted drying and freeze-drying are significantly more gentle compared to air drying in an oven [42]. Considering carotenoids in sea fennel, Sarrou et al. [74] identified lutein and neoxanthin as major carotenoid compounds.

### 2.4. Antioxidant Properties

**Figure 3:**
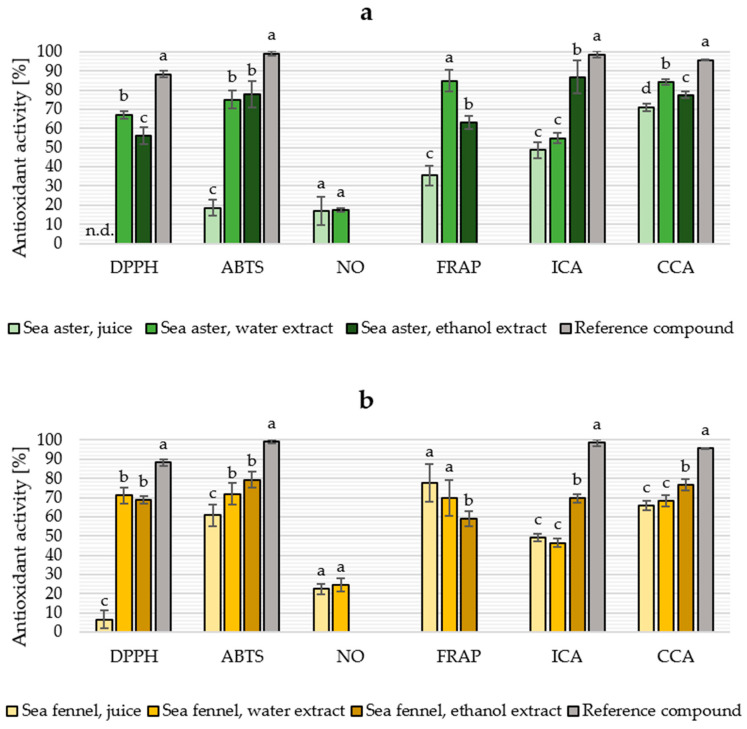
Antioxidant activity [%] of the 10 mg/mL sea aster (*Tripolium pannonicum*, (**a**)) and sea fennel (*Crithmum maritimum*, (**b**)) juice and extracts in relation to negative control. The reference compounds are gallic acid 1 mg/mL (DPPH and ABTS) and ethylenediaminetetraacetic acid 1 mg/mL (ICA and CCA). Different letters denote significantly different results, calculated individually for each assay. DPPH: 2,2-diphenyl-1-picrylhydrazyl, ABTS: 2,2′-azinobis-(3-ethylbenzothiazoline-6-sulfonic acid), NO: nitric oxide, FRAP: ferric reducing antioxidant power, ICA: iron chelating activity, CCA: copper chelating activity, n.d.: no activity detected. Different letters denote significantly different results (*p* ≤ 0.05), calculated individually for each assay and plant species.

The antioxidant activity of sea aster and sea fennel juice fractions and extracts is presented in Figure 3. The juice faction from sea fennel exhibited high ABTS scavenging activity (60.75%) and FRAP (77.67%), and CCA (71.19%) in 10 mg/mL concentration; however, the half-maximum effective concentration (EC_50_) values were higher compared to extracts (Table 4). The DPPH and ABTS scavenging activity of both sea fennel and sea aster aqueous extracts were similar, but sea aster had more pronounced antioxidant activity in metal-based assays. On the contrary, sea fennel fractions had higher NO scavenging activity, estimating the extract’s anti-inflammatory properties, than sea aster fractions. Especially in ethanol extracts, there was a large variation in the antioxidant activity between different assays, highlighting the importance of running multiple assays to evaluate the extracts’ bioactivity as a single assay provides only a limited view of the antioxidant potential. The results from the total contents of different phenolic groups also cannot directly be reflected in the antioxidant activities, bringing interest to specific phytochemicals or potential synergic interactions between them. The ethanol-soluble samples turned cloudy during the NO radical scavenging assay when the Griess reagent was added; therefore, it was not possible to measure the activity.

Pereira et al. [34] reported high DPPH and ABTS scavenging activity (>86%), moderate NO scavenging activity (>37%), and high FRAP (>98%) of aqueous sea fennel extracts in low concentrations (“cup-of-tea” infusion and decoction), whereas the ICA and CCA were lower than the results obtained in this study. Moreover, Pedreiro et al. [58] reported EC_50_ values of 36.5 μg/mL and 37.3 μg/mL for DPPH and ABTS scavenging activity for aqueous sea fennel infusion. Those results are nearly a hundred-fold lower than the results obtained in this study. Siracusa et al. [75] also reported a 88% DPPH scavenging activity of 0.4 mg/mL sea fennel infusion with potential relation to high chlorogenic acid content. The differences in results could be explained by the plant growth stage and fractions used but also growth conditions, as cultivated plants have previously been shown to have lower phytochemical concentration and antioxidant activity compared to wild plants due to controlled conditions with less pronounced abiotic stresses [64,76].

Whereas the antioxidant activity of different sea fennel fractions is covered in literature, the use of sea aster for health-promoting purposes is rather unexplored. Stankovic et al. [67] reported an EC_50_ value of 0.13 mg/mL for the DPPH scavenging activity of sea aster methanol extract, which is significantly lower compared to the obtained results in this study. On the other hand, Wubshet et al. [40] used nuclear magnetic resonance technology and found caffeoyl esters, flavonoids, and flavonoid glycosides in sea aster extract, which they described as potential compounds to explain the ABTS scavenging activity.

Exposure to excessive reactive oxygen species (ROS) and following oxidative stress has been linked to the development and progress of various health issues, such as neurodegenerative, metabolic and autoimmune diseases and cancer, often due to their multi-factorial nature [77,78]. Furthermore, as the individual’s nutrition, level of exercise, and exposure to toxins significantly contribute to the production of ROS, many of the related issues are called “lifestyle diseases” [77]. Therefore, antioxidant products capable of preventing or alleviating these emerging conditions are often targets of bioprospecting. Due to their active properties, botanical extracts are common ingredients in various high-value products, for example, in the nutraceuticals and cosmetics sectors. However, identifying and concentrating or separating compounds responsible for specific activities could be desired for some applications, such as biomedicines. Bioguided fractionation is a potential analysis method to find the target compounds. Resin absorption, chromatography, and different filtration methods are examples of technologies studied to purify and isolate compounds from extracts and separate compounds from the green juice fraction in green biorefineries [51,79,80].

### 2.5. Enzyme Inhibition

**Figure 4:**
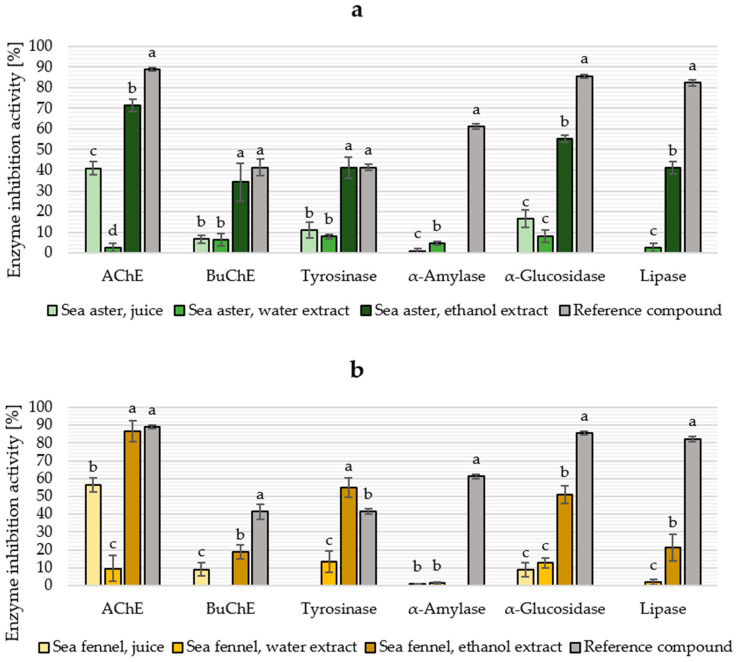
Enzyme inhibition activity of 10 mg/mL sample solutions from sea aster (*Tripolium pannonicum*, (**a**)) and sea fennel (*Crithmum maritimum*, (**b**)) juice and extracts. Reference compounds: galantamine 1 mg/mL (AChE and BuChE), arbutin 1 mg/mL (tyrosinase), acarbose 10 mg/mL (α-amylase and α-glucosidase), and orlistat 1 mg/mL (lipase). Different letters denote significantly different results, calculated individually for each assay. AChE: acetylcholinesterase, BuChE: butyrylcholinesterase. Different letters denote significantly different results (*p* ≤ 0.05), calculated individually for each assay and plant species.

Inhibition activity against enzymes linked to different diseases is summarized in Figure 4. It was not possible to determine the inhibition activity against butyrylcholinesterase (BuChE) for sea fennel water extract, tyrosinase for sea fennel juice, or lipase for juice fractions from both species due to instability issues with the assays. The inhibition of α-amylase was visually observed in ethanol-based samples. However, due to sample precipitation, it was not possible to reliably measure the absorbance and determine the activity. In general, the enzyme inhibition activity was higher in ethanol extracts compared to juice fractions or aqueous extracts.

For sea aster ethanol extract, the acetylcholinesterase (AChE) inhibition activity (71.5%) is higher than previously reported for ethanol extracts (concentration not disclosed) from other Asteraceae species [81], and BuChE inhibition activity (34.2%) was the highest from the tested samples. To the best of our knowledge, this is the first study to report the AChE and BuChE inhibition activity of sea aster fractions. Aghraz et al. [82] determined the neuroprotective properties of 0.4 mg/mL infusions from two other halophytes from the Asteraceae family and shower moderate and low inhibition activities against AChE and BuChE, respectively, comparable to obtained results for the sea aster juice fraction.

In sea fennel samples, the AChE inhibition activity of ethanol extract (86.7%) was significantly higher, and the inhibition of BuChE is nearly the same compared to the study by Mekinić et al. [37], who reported 27.2% and 19.6% inhibition of AChE and BuChE, respectively, using sea fennel ethanol extract (concentration not disclosed). Nguir et al. [83] reported 31.2% AChE inhibition activity of 1 mg/mL sea fennel essential oils; indeed, many studies of sea fennel bioactivity focus on the essential oil fraction. The juice fraction from both species exhibited higher inhibition of AChE compared to aqueous extracts, indicating that the compounds potentially responsible for the activity are mostly ethanol-soluble or free water-soluble compounds, which end up in the juice fraction in the initial fractionation process.

Tyrosinase inhibitors have a role in the food, cosmetics, and pharmaceutical industries. As a type of polyphenol oxidase, tyrosinase causes an undesired browning in plant-derived food products [84]. In animals, tyrosinase produced in melanocytes participates in melanin production, and tyrosinase inhibitors are used in cosmetics as skin-whitening agents [85,86] and have increased interest in the treatment of skin disorders with hyperpigmentation and melanoma skin cancer [23]. Tested ethanol extracts exhibited moderate tyrosinase inhibition at 10 mg/mL, 41.2% and 55.1% for sea aster and sea fennel, respectively. Calvo et al. [87] studied the potential of 50% aqueous ethanol halophyte extracts to prevent melanosis in shrimp, and they reported both sea fennel and sea aster to have diphenol oxidase inhibition activity of 30–40% at 10 mg/mL. Sea fennel cell culture has already been commercialized as an active cosmetic ingredient due to its tyrosinase-inhibitory properties [88]. The 50% ethanol extracts from halophyte species *Inula crithmoides* and *Daucus carota*, belonging to Asteraceae and Apicaceae families, respectively, have also previously shown tyrosinase inhibition activity; major phenolic compounds in the extracts are chlorogenic acid and rutin for *Inula crithmoides*, and chlorogenic acid and quercetin for *Daucus carota* [23].

There is a limited amount of existing literature considering the anti-diabetic and lipase-inhibitory properties of raw extracts from sea fennel and sea aster. Wubshet et al. [40] found quercitin and kaempferol derivatives from the sea aster to be present in the extract fraction with α-glucosidase activity. Souid et al. [24] found chlorogenic acid, neochlorogenic acid, and quercitin derivates to be the most abundant phenolics in 80% aqueous ethanol sea fennel extract, and these phytochemicals have been described as potential anti-diabetic compounds [89,90,91]. These compounds inhibit α-amylase and α-glucosidase, the key enzymes responsible for the digestion of carbohydrates, delaying and limiting glucose absorption and potentially preventing hyperglycemia in people with type II diabetes mellitus [90,91]. Obesity has been shown to increase an individual’s risk of a multitude of health concerns, and one of the suggested treatments to control the excessive accumulation of body fat has been to inhibit the activity of pancreatic lipase, an enzyme with a key role in fat metabolism [92]. Phytochemicals, such as flavonoids, alkaloids, saponins and terpenoids, have been shown to have lipase inhibition activity [93]. Ethanol extract of sea aster exhibited moderate lipase inhibition at 10 mg/mL, and this sample also exhibited the highest content of TFC, whereas other tested extracts showed only low lipase inhibition activity. Extracts from other medicinal halophytes have previously been reported to have lipase inhibition with half maximal inhibitory concentrations of 0.16 mg/mL and 1.33 mg/mL for *Limonium sinuatum* and *Lobularia maritima* methanolic extracts, respectively, and 41.7% lipase inhibition at 10 mg/mL with ethanol extract from *Salicornia ramosissima* [94,95,96]. The anti-obesity properties of sea fennel tincture have also been studied considering the increased energy expenditure (thermogenic drug), but no positive effects were observed [97].

Extracting phytochemicals for medicinal purposes and targeting chronic diseases with plant-derived products has attracted attention in recent years [21,68,77,98]. The bioprospecting of novel feedstocks is beneficial for finding new potential treatments for health concerns. Targeting products for biomedical and nutraceutical applications is also important for the economic feasibility of integrated biorefineries and sustainable bioeconomies. The results show that extracts from residual halophyte fraction can have similar or higher biological activity as extracts from fresh food-grade plants, highlighting the potential of the residual fractions for full feedstock valorization. In tested samples, the highest bioactivities in enzyme inhibition assays were observed in ethanol extracts. In large-scale biorefinery applications, minimizing the use of organic solvent is often desired, and the optimization of the extraction method is key in the process development; however, each method has advantages and disadvantages related to extraction efficiency, chemical use and toxicity, and energy consumption [51,68,94].

## 3. Materials and Methods

### 3.1. Raw Material

Plant material was obtained from the Institute of Botany of Leibniz University Hannover, where it was grown in a greenhouse. Halophytes were harvested when the plants were fresh but partially lignified (non-food grade). Sea aster was cultivated in a hydroponic system under 120 mM NaCl salinity in similar conditions as previously described by Hulkko et al. [31] and harvested at the vegetative growth stage in June 2021. Sea fennel was cultivated in non-saline conditions in pots with sandy soil and harvested at the flowering stage in September 2021. At the harvest stage, the biomass was partly lignified and had developed a fibrous texture. Biomass was frozen after harvesting and kept in the freezer at −24 °C before further processing.

### 3.2. Biomass Fractionation and Extraction

Both biomasses were fractionated to green juice and fiber residue using a pilot-scale double-auger juicer. Green juice fractions were centrifuged and filtered (Whatman, GE Healthcare, Chicago, IL, USA), concentrated using a rotary evaporator, freeze-dried (ModulyoD, Thermo Scientific, Waltham, MA, USA), and re-solubilized to distilled water with a concentration of 10 mg/mL. Fiber residue fractions were dried overnight in a 60 °C fan oven(Memmert, Schwabach, Germany), size-reduced by knife-milling to particle size < 2 mm, and stored at room temperature (RT) protected from the light before analysis. The dry matter (DM), ash, and carbohydrate content of the fractions were determined using analytical protocols by National Renewable Energy Laboratory [99,100,101]. For structural carbohydrates and Klason lignin, the analysis was run in duplicates for samples and recovery standards. Separated sugars were glucose, xylose and arabinose, and samples were analyzed using high-performance liquid chromatography (126 Infinity II, Agilent Technologies, Santa Clara, CA, USA) with 5 mM H_2_SO_4_ as mobile phase, organic acid column (Aminex HPX-87H, Bio-Rad Laboratories, Hercules, CA, USA) at 63 °C, and refractive index detector at 30 °C.

Plant extracts were obtained from fiber residue fraction using conventional Soxhlet apparatus with a 100 mL extraction chamber and 250 mL of different solvents, namely: demineralized water, 70% aqueous ethanol, and n-hexane. All extractions were run as parallel experiments. For water and ethanol extractions, samples (10 g) were extracted for 8 h, and for n-hexane extraction, samples (5 g) were extracted for 6 h. The excess solvent was recovered at 40 °C using a rotary evaporator combined with a vacuum pump (KNF, Stockholm, Sweden). Obtained water and ethanol extracts were freeze-dried and re-solubilized to the corresponding solvents at a concentration of 10 mg/mL.

### 3.3. Transesterification and Determination of Fatty Acids

FA present in the n-hexane extracts were converted into fatty acid methyl esters (FAME) by transesterification. A small amount of obtained lipids from the n-hexane extraction (0.15 g) was dissolved in 0.5 M methanolic sodium hydroxide (1 mL) at 90 °C. Samples were cooled to RT, boron trifluoride (1 mL) and hydroquinone solution (0.5 mL) was added, and samples were heated at 90 °C for 5 min. Phase separation was achieved using saturated NaCl aqueous solution (4 mL) and n-heptane (3 mL). After separation, the n-heptane soluble fraction was recovered. FA profile was determined using gas chromatography (Clarus 500, Perkin Elmer, Waltham, MA, USA), capillary column (Elite-WAX, 30 m × 0.25 mm ID × 0.25 μm, Perkin Elmer, Waltham, MA, USA) and helium as carrier gas. The temperature program was set to 1 min at 150 °C, heating 10 °C/min until 240 °C and keeping it at 240 °C for 10 min. Compounds were detected with mass spectrometry, and the results were compared to the National Institute of Standards and Technology (NIST) library and given as relative concentrations.

### 3.4. Total Photosynthetic Pigments

The total concentrations of chlorophylls (CHL) and carotenoids (TCA) were determined in green juice fractions and ethanol extracts by spectrophotometry. The absorbance was measured at 470 nm, 649 nm, and 665 nm using a UV-visible spectrophotometer (Genesys 50, Thermo Scientific, Waltham, CA, USA) with a quartz cuvette. Turbidity at 750 nm was considered. Calculations were carried out using the formulas described by Lichtenthaler and Wellburn [102]:

CHL a = 13.95 × A_665_ − 6.88 × A_649_, (1)

CHL b = 24.96 × A_649_ − 7.32 × A_665_, (2)

TCA = (1000 × A_470_ − 2.05 × CHL a − 114.8 × CHL b)/245 (3)

### 3.5. Phenolic Compounds in Plant Fractions

The total contents of different phenolic compound groups were determined in juice fractions, and water and ethanol extracts. TPC were determined as the amount of gallic acid equivalent (GAE) in the juice or extract samples by the Folin–Ciocalteau (F-C) assay described by Velioglu et al. [103] adapted to 96-well plates. Samples (5 μL) were mixed with F-C reagent in ethanol (100 μL) and incubated in RT for 10 min, after which 75 g/L aqueous sodium carbonate (100 μL) was added, and plates were further incubated for 90 min. The calibration curve (*R*^2^ = 0.997) was made using gallic acid, and absorbance was read at 650 nm using a microplate reader (EZ Read 400, Biochrom, Cambridge, UK).

TFC were determined using a method by Pirbalouti et al. [104] by mixing samples (50 µL) with 2% aluminum chloride in methanol solution (50 µL), incubating plates for 10 min at RT, and reading the plates at 405 nm. Quercetin was used as a standard for calibration (*R*^2^ = 0.999), and results are expressed as the amount of quercetin equivalent (QE) in the mass unit of the dried sample.

TCT, also known as proanthocyanidins, were determined using the assay by Li et al. [105] with p-dimethylaminocinnamaldehyde hydrochloric acid (DMACA-HCl). Juice and extract samples (10 μL) were mixed with 1% DMACA in methanol (200 μL) and 37% HCl (100 μL). Plates were incubated for 15 min at RT, and absorbance was read at 640 nm. Results are expressed as catechin equivalent (CE) in the dry extract and calculated based on the calibration curve (*R*^2^ = 0.991).

TAC were determined as described by Mazza et al. [106] by mixing an aliquot of the samples (20 µL) with 95% ethanol containing 0.1% HCl (20 µL) and 1 M HCl (160 µL). The absorbance was read at 492 nm, and the results are expressed as cyanidin chloride equivalent (CCE) based on the calibration curve (*R*^2^ = 0.991).

### 3.6. In Vitro Radical Scavenging Activity

RSA of juice and water and ethanol extracts was tested using colorimetric methods towards the following radicals: DPPH, ABTS, and NO. Samples were tested at 10 mg/mL concentrations, absorbances were read using a microplate reader, and RSA was calculated as a percentage relative to a negative control sample (blank). Gallic acid at a 1 mg/mL concentration was used as a positive control in all RSA assays. For the DPPH assay by Brand-Williams et al. [107], samples (22 µL) were mixed with 120 µM DPPH in ethanol (200 µL), incubated for 30 min in the dark, and reading the absorbance at 492 nm.

The ABTS scavenging assay was carried out as described by Re et al. [108]. First, the ABTS^•+^ solution with a molarity of 7.4 mM was obtained by mixing ABTS (100 mg) with 2.6 mM potassium persulphate (100 mL) in RT in the dark and incubating the mixture overnight. Afterward, the ABTS^•+^ solution was diluted with ethanol until an absorbance of approximately 0.7 at 734 nm was achieved. This diluted solution was used in the assay, where samples (10 µL) were mixed with ABTS^•+^ solution (190 µL) and incubated for 6 min in the dark. The absorbance was read at 650 nm.

The NO scavenging assay was carried out by the method described by Baliga et al. [109]. First, samples (50 µL) were mixed with 10 mM sodium nitroprusside (50 µL) in 96-well plates and incubated for 90 min in RT. Afterward, Griess reagent (50 µL) was added, and plates were read at 562 nm.

### 3.7. In Vitro Antioxidant Activity by Metal-Based Assays

Metal-based assays were also carried out using juice and water and ethanol extract samples at a concentration of 10 mg/mL. Results are expressed as a percentage relative to a negative control sample. The capability to bind and hold iron and copper was analyzed with chelating assays described by Megías et al. [110], and ethylenediaminetetraacetic acid (EDTA) with a concentration of 1 mg/mL was used as a positive control. In the ICA assay, samples (30 µL) were mixed with water (200 µL) and 0.01% aqueous FeCl_2_ (30 µL) in 96-well plates. Plates were incubated for 30 min, and after incubation, 40 mM aqueous ferrozine (12.5 µL) was added. Plates were further incubated for 10 min, and absorbance was read at 562 nm. In the CCA assay, samples (30 µL) were mixed with 50 mM sodium acetate buffer (200 µL), 0.005% aqueous CuSO_4_ (100 µL) and 4 mM aqueous pyrocatechol violet (6 µL), and plates were immediately read at 620 nm.

The FRAP assay was performed according to Megías et al. [110] by mixing equal parts (50 µL) of samples, distilled water, and 1% potassium ferrocyanide in 96-well plates and incubating for 20 min at 50 °C. Afterward, 10% aqueous trichloroacetic acid (50 µL) and 0.1% aqueous FeCl_3_ (10 µL) were added, plates were incubated for 10 min at RT, and absorbance was read at 650 nm. Gallic acid at 1 mg/mL concentration was used as a positive control.

### 3.8. In Vitro Enzyme Inhibition Assays

Inhibition activity against enzymes related to different diseases was tested in vitro using green juice fractions and water and ethanol extracts at a concentration of 10 mg/mL. Results were expressed as the percentage of inhibition related to a negative control sample. Potential anti-diabetic properties of halophyte juices and extracts were determined by measuring the inhibition of α-amylase and α-glucosidase. Acarbose, a known anti-diabetic drug on the market, was used as a reference at a concentration of 10 mg/mL. For α-amylase inhibition activity, an assay developed by Xiao et al. [111] was used, which is based on the reaction between iodine solution and starch. Equals parts (40 µL) of samples, 0.1% boiled potato starch solution, and 100 U/mL α-amylase in 0.1 M sodium phosphate buffer solution (pH 6.9) were mixed in 96-well plates and incubated at 37 °C for 10 min. Afterward, 1 M HCl (20 µL) and iodine solution (100 µL), consisting of 5 mM I_2_ and 5 mM KI in distilled water, were added, and absorbance was read at 570 nm. A negative control sample with no enzyme (100% inhibition, blank) was used in the basis of calculation, whereas a control sample with an enzyme was used for color correction. Results were calculated as follows:

α-amylase inhibition activity [%] = (A_570_ sample − A_570_ color control)/A_570_ blank × 100% (4)

In the α-glucosidase inhibition activity assay described by Custódio et al. [112], samples (50 µL) were mixed with 1 U/mL *Saccharomyces cerevisiae* α-glucosidase in phosphate buffer (100 µL, pH 7.0), and plates were incubated at 25 °C for 10 min. After incubation, 5 mM p-nitrophenyl-α-d-glucopyranoside (50 µL) was added, and plates were incubated for a further 5 min at 25 °C before reading the absorbance at 405 nm.

The ability to inhibit tyrosinase was tested as described by Trentin et al. [113] by mixing samples (70 µL) with 333 U/mL tyrosinase solution (30 µL) in 25 mM potassium phosphate buffer (pH 6.5) and incubating for 5 min at RT. After incubation, a substrate solution (110 µL) of 2 mM L-tyrosine diluted in buffer was added, and plates were incubated for 30–45 min in RT before reading the absorbance at 405 nm. Arbutin, a compound known to inhibit tyrosinase and prevent melanin formation, was used as a reference at a concentration of 1 mg/mL.

Inhibition of AChE and BuChE was measured using the method by Ellman et al. [114] by mixing samples (20 µL) with 0.02 M sodium phosphate buffer (140 µL, pH 8.0) and 0.28 U/mL AChE or BuChE enzyme solution in pH 7.0 buffer (20 µL), respectively. Plates were incubated for 15 min at 25 °C, and afterward, acetylcholine iodide or butyrylcholine iodide, respectively, in 4 mg/mL pH 8.0 buffer solution (10 µL) was added. Then, Ellman’s reagent (5,5′-dithiobis(2-nitrobenzoic acid)) in 1.2 mg/mL ethanol (20 µL) was added. Samples were incubated for another 15 min at 25 °C, and absorbance was read at 405 nm. Galantamine, a drug used to treat dementia, was used as a reference at 1 mg/mL concentration.

Lipase inhibition activity was measured using a protocol based on that described by McDougall et al. [115]. Samples (20 µL) were mixed with 100 mM Tris-HCl buffer (200 µL, pH 8.2), 1 mg/mL porcine pancreatic lipase in buffer solution (20 µL), and 5.1 mM 4-nitrophenyl dodecanoate in ethanol (20 µL). Plates were incubated at 37 °C for 10 min before reading the absorbance at 405 nm. Orlistat, a medicinal compound used to support weight loss, was used as a reference at a 1 mg/mL concentration.

### 3.9. Statistical Methods

Results are given as the mean values with standard deviation marked in brackets unless stated otherwise. The number of replicates was (*n* = 6) for analysis run in microplates and (*n* = 3) for other analyses unless stated otherwise. One-way analysis of variance (ANOVA) and Tukey honest significant difference (HSD) test were run to evaluate differences between the results, and significantly different results are marked with different letters. For the antioxidant activities, the half-maximum effective concentration (EC_50_) was calculated using an online tool by AAT Bioquest Inc. (Pleasanton, CA, USA) [116] when the obtained activity at 10 mg/mL concentration was higher than 50%.

## 4. Conclusions

The chemical composition and biological activities of the juice and fiber residue extracts from sea aster and sea fennel after fractionation with screw-press were analyzed to evaluate the potential of non-food grade residuals to produce high-value, low-volume bioproducts as a part of halophyte-based integrated biorefinery. Analyzed plant fractions exhibited interesting properties for potential added value creation of residual biomass, such as antioxidant activity and the inhibition of enzymes related to chronic diseases. Halophytes from biosaline agriculture could provide not only a healthy food source but also a feedstock to produce extracts for biomedicines, nutraceuticals, and cosmetics. Process development and optimization, as well as phytochemical analysis and bioavailability assays, are needed to maximize the potential and valorization of halophyte biomass. Overall, sea aster and sea fennel can be seen as interesting species for further biorefinery investigations, and in general, expanding the utilization of halophytes could provide an important contribution to sustainable bioeconomies.

## Figures and Tables

**Table 1:** Dry matter (DM) content and composition of halophyte fractions and extraction yields. Values are expressed as means, with the standard deviation marked in brackets.

Fraction	DM [*w*/*w* %]	Water Extract [g/100 gDM]	Ethanol Extract [g/100 gDM]	n-hexane Extract [g/100 gDM]	Sugars [g/100 gDM]	Lignin [g/100 gDM]	Ash [g/100 gDM]
**Sea aster (*Tripolium pannonicum*)**							
Juice	4.01 (0.04)	n/a	n/a	n/a	22.98 (0.71)	n/a	47.71 (0.76)
Fibres	n/a	18.73 (0.50)	25.88 (2.47)	2.87 (0.04)	37.64 (4.56)	16.98 (1.18)	6.30 (0.01)
**Sea fennel (*Crithmum maritimum*)**							
Juice	7.26 (0.01)	n/a	n/a	n/a	13.24 (3.49)	n/a	36.56 (0.09)
Fibres	35.67 (0.84)	16.54 (0.33)	13.56 ^1^	2.20 (0.10)	34.62 (0.67)	18.76 (4.78)	10.19 (0.07)

^1^ Due to the small amount of biomass, it was not possible to run the analysis in triplicate and provide the standard deviation.

**Table 2:** Relative concentration of fatty acids (FA) in n-hexane extracts obtained from fiber residues of sea aster (*Tripolium pannonicum*) and sea fennel (*Crithmum maritimum*). Values are expressed as means, with the standard deviation marked in brackets.

Fatty Acids	Sea Aster [% FA/Total FA]	Sea Fennel [% FA/Total FA]
Myristic acid	n.d.	1.5 (2.2)
Palmitic acid	19.0 (0.2)	28.9 (1.8)
Palmitoleic acid	n.d.	1.9 (2.7)
Stearic acid	1.6 (0.0)	6.3 (0.3)
Oleic acid	1.2 (0.5)	11.5 (0.2)
Linoleic acid	24.9 (0.2)	34.4 (1.8)
α-Linolenic acid	53.2 (0.3)	15.5 (0.7)
Arachidic acid	n.d.	n.d.
Behenic acid	n.d.	n.d.
Lignoceric acid	n.d.	n.d.
Σ SFA	20.6 (0.2)	36.7 (4.3)
Σ MUFA	1.2 (0.5)	13.4 (2.9)
Σ PUFA	78.2 (0.5)	49.9 (1.2)
ω-6/ω-3	0.5 (0.0)	2.2 (0.2)

n.d.: not detected, SFA: saturated fatty acids, MUFA: monounsaturated fatty acids, PUFA: polyunsaturated fatty acids.

**Table 3:** Dry matter (DM) content and composition of halophyte fractions and extraction yields. Values are expressed as means, with the standard deviation marked in brackets. Different letters denote significantly different results (*p* ≤ 0.05), calculated individually for each compound group.

Fraction	TPC [mgGAE/gDM]	TFC [mgQE/gDM]	TCT [mgCE/gDM]	TAC [mgCCE/gDM]	CHL ^a^ [µg/gDM]	CHL ^b^ [µg/gDM]	TCA [µg/gDM]
**Sea aster (*Tripolium pannonicum*)**							
Juice	n.d.	n.d.	n.d.	n.d.	89.42 (14.56) ^c^	148.30 (24.23) ^c^	125.75 (8.85) ^c^
Water extract	32.34 (6.80) ^c^	5.41 (0.68) ^ab^	n.d.	4.37 (0.74) ^a^	n/a	n/a	n/a
Ethanol extract	45.20 (5.27) ^b^	6.58 (1.49) ^a^	0.43 (0.60)	n/a	2614.08 (12.19) ^a^	1017.87 (17.94) ^a^	299.05 (3.79) ^a^
**Sea fennel (*Crithmum maritimum*)**							
Juice	14.97 (4.56) ^d^	n.d.	n.d.	n.d.	13.62 (0.69) ^d^	44.28 (0.73) ^d^	85.34 (3.20) ^d^
Water extract	33.53 (2.53) ^c^	4.90 (0.81) ^b^	n.d.	1.90 (0.21) ^b^	n/a	n/a	n/a
Ethanol extract	64.70 (9.01) ^a^	1.84 (1.15) ^c^	n.d.	n/a	1008.44 (14.74) ^b^	430.92 (9.31) ^b^	261.79 (2.03) ^b^

n.d.: not detected or concentration lower than the limit of detection, n/a: not available, TPC: total phenolic compounds [mgGAE/gDM], TFC: total flavonoids [mgQE/gDM], TCT: total condensed tannins [mgCE/gDM], TAC: total anthocyanins [mgCCE/gDM], CHL: chlorophyll [µg/gDM], TCA: total carotenoids [µg/gDM], GAE: gallic acid equivalent, QE: quercitin equivalent, CE: catechin equivalent, CCE: cyanidin chloride equivalent, DM: dry matter.

**Table 4:** The half-maximum effective concentrations (EC_50_) [mg/mL] of antioxidant activity of juice and fiber residue extracts from sea aster (*Tripolium pannonicum*) and sea fennel (*Crithmum maritimum*).

Fraction	DPPH	ABTS	NO	FRAP	ICA	CCA
**Sea aster (*Tripolium pannonicum*)**						
Juice	n.d.	>10	>10	>10	>10	3.18
Water extract	<10	2.24	>10	1.91	<10	3.40
Ethanol extract	7.67	4.86	n/a	1.66	1.15	2.37
**Sea fennel (*Crithmum maritimum*)**						
Juice	>10	4.59	>10	3.46	>10	6.79
Water extract	3.53	4.36	>10	1.13	>10	7.03
Ethanol extract	2.84	3.95	n/a	<10	3.10	3.32

DPPH: not detected, 2,2-diphenyl-1-picrylhydrazyl, ABTS: 2,2′-azinobis-(3-ethylbenzothiazoline-6-sulfonic acid), NO: nitric oxide, FRAP: ferric reducing antioxidant power, ICA: iron chelating activity, CCA: copper chelating activity, n.d.: no activity detected, n/a: not available.

## Data Availability

The data generated during the study are available from the corresponding author on request.
